# ﻿Morphological and molecular analyses reveal two new species of *Microcera* (Nectriaceae, Hypocreales) associated with scale insects on walnut in China

**DOI:** 10.3897/mycokeys.98.103484

**Published:** 2023-05-29

**Authors:** Feng Liu, Yu Deng, Fei-Hu Wang, Rajesh Jeewon, Qian Zeng, Xiu-Lan Xu, Ying-Gao Liu, Chun-Lin Yang

**Affiliations:** 1 College of Forestry, Sichuan Agricultural University, Chengdu, Sichuan, 611130, China; 2 National Forestry and Grassland Administration Key Laboratory of Forest Resources Conservation and Ecological Safety on the Upper Reaches of the Yangtze River and Forestry Ecological Engineering in the Upper Reaches of the Yangtze River Key Laboratory of Sichuan Province, College of Forestry, Sichuan Agricultural University, Chengdu, Sichuan, China; 3 Department of Health Sciences, Faculty of Medicine and Health Sciences, University of Mauritius, Reduit, Mauritius; 4 Forestry Research Institute, Chengdu Academy of Agricultural and Forestry Sciences, Chengdu 611130, Sichuan Province, China

**Keywords:** Two new taxa, entomopathogenic fungi, morphology, phylogenetic analyses

## Abstract

The fungal genus *Microcera* consists of species mostly occurring as parasites of scale insects, but are also commonly isolated from soil or lichens. In the present study, we surveyed the diversity and assess the taxonomy of entomopathogenic fungi in Sichuan Province, China. Two new species of *Microcera*, viz. *M.chrysomphaludis* and *M.pseudaulacaspidis*, were isolated from scale insects colonising walnut (*Juglansregia*). Maximum Likelihood and Bayesian Inference analyses of ITS, LSU, *tef*1-α, *rpb*1, *rpb*2, *acl*1, *act*, *tub*2, *cmd*A and *his*3 sequence data provide evidence for the validity of the two species and their placement in Nectriaceae (Hypocreales). *Microcerapseudaulacaspidis* primarily differs from similar species by having more septate and smaller cylindrical macroconidia, as well as DNA sequence data. Meanwhile, *Microcerachrysomphaludis* has elliptical, one-septate ascospores with acute ends and cylindrical, slightly curved with 4–6 septate macroconidia up to 78 µm long. Morphological descriptions with illustrations of the novel species and DNA-based phylogeny generated from analyses of multigene dataset are also provided to better understand species relationships.

## ﻿Introduction

The genus *Microcera* Desm. (Nectriaceae, Hypocreales) was introduced in the 19^th^ century and was typified by *M.coccophila* Desm., commonly known as the “red-headed fungus”. *Microcera* has been considered to be a synonym of the *Fusarium* Link in some major taxonomic revisions (Booth 1971; Nelson et al. 1983; Leslie and Summerell 2006). The genus is characterised by superficial, flame-like conidiomata, forming a fusarium-like asexual stage (Gräfenhan et al. 2011; Herrera et al. 2013). *Microcera* species exhibit diverse ecological characteristics and are typically regarded as entomogenous fungi that are associated with scale insects, although they can occasionally be isolated from other substrates, such as aphids, adelgids, lichens and soil (Gräfenhan et al. 2011; Crous et al. 2021a, b, 2022a).

Currently, there are eight accepted species within the genus *Microcera* (Bills et al. 2009; Gräfenhan et al. 2011; O’Donnell et al. 2012; Herrera et al. 2013; Dao et al. 2015, 2016; Lombard et al. 2015; Crous et al. 2021b, 2022a; Xu et al. 2021). Based on DNA sequence data and ecological association, Gräfenhan et al. (2011) revised many anamorph- and teleomorph-typified genera of the Nectriaceae, resurrected *Microcera* and accepted four *Microcera* species, viz., *M.coccophila*, *M.diploa* (Berk. & M.A. Curtis) Gräfenhan & Seifert, *M.rubra* Gräfenhan & Seifert and *M.larvarum* (Fuckel) Gräfenhan, Seifert & Schroers. Lombard et al. (2015) supported *Microcera* as a monophyletic group distantly related to *Fusarium*, based on further phylogenetic inferences from DNA sequence data. Xu et al. (2021) isolated *M.kuwanaspidis* X.L. Xu & C.L. Yang from armoured scale insects *Kuwanaspishowardi* on *Phyllostachysheteroclada* in China. Two additional species, *M.lichenicola* and *M.physciae* Crous & Boers have been described from lichens (Crous et al. 2021b, 2022a).

During a survey of entomopathogenic fungi in Sichuan Province, China, two *Microcera* species, in association with the two scale insects *Pseudaulacaspispentagona* and *Chrysomphalusaonidum* on walnut, were isolated. *Microcerapseudaulacaspidis* sp. nov. and *M.chrysomphaludis* sp. nov. are introduced here based on the morphological characteristics and multi-locus analyses (DNA based). They were compared morphologically with existing taxa. In this study, comprehensive descriptions, micrographs of macroscopic and microscopic morphological characteristics, as well as DNA sequence data, are provided to support the establishment of the new species.

## ﻿Materials and methods

### ﻿Specimen collection and isolation

Three specimens of scale insects (SICAU 22-0161, SICAU 22-0162 and SICAU 22-0163) that were infected, were collected from Neijiang City (29°48′15″N, 105°06′44″E) and Liangshan Yi Autonomous Prefecture (26°56′43″N, 102°16′16″E), Sichuan Province, on 16 April and 8 October 2022. The specimens were placed in sterilised tubes or plastic boxes and returned to the laboratory as described by Senanayake et al (2020). The fungi were isolated, based on the single spore isolation technique described by Chomnunti et al. (2014). Cultures were grown on PDA for 20–40 days, at 25 °C, under 12 h light/12 h dark for recording growth rates, shape, texture and colour of the colonies. Ascomata and sporodochia were observed and photographed using a dissecting microscope NVT-GG (Shanghai Advanced Photoelectric Technology Co. Ltd., Shanghai, China). We observed microscopic characteristics, such as asci, ascospores, pseudoparaphyses, ascomata wall, conidia, conidiophores, number of septa, metulae and conidiophores using an Olympus BX43. No fewer than 20 measurements of the two species were made for each feature using the Image Frame Work (IFW 0.9.0.7). The type specimens were deposited at the Herbarium of Sichuan Agricultural University, Chengdu, China (SICAU). The ex-type cultures were deposited at the Culture Collection in Sichuan Agricultural University (SICAUCC).

### ﻿DNA extraction, PCR amplification and nucleotide sequencing

The New Plant Genomic DNA Kit (Beijing Aidlab Biotechnologies Co., Ltd, Beijing, China) was used to extract genomic DNA from fresh fungal mycelium. The extracted DNA to be used was stored at -20 °C. Amplified gene markers and their corresponding primers are shown in Table 1. Polymerase chain reaction (PCR) was performed in 25 µl reaction mixture containing 22 µl Master Mix (Beijing LABLEAD Biotech Co., Ltd., Beijing, China), 1 µl DNA template and 1 µl each of forward and reverse (10 µM) primers. The amplification reactions were performed as described by Gräfenhan et al. (2011), Lombard et al. (2015), Dai et al. (2016) and Wanasinghe et al. (2021). PCR products were sequenced at Hangzhou Youkang Biotech Co., Ltd., Chengdu, China. The newly-generated sequences were deposited in GenBank. New species are established as recommended by Jeewon and Hyde (2016).

**Table 1.** Gene markers and primer pairs used in this study.

Gene markers	Primers	Sequences of Primers 5’-3’	References
*acl*1	acl1-230up	AGCCCGATCAGCTCATCAAG	Gräfenhan et al. (2011)
	acl1-1220low	CCTGGCAGCAAGATCVAGGAAGT	
*act*	ACT-512F	ATGTGCAAGGCCGGTTTCGC	Carbone and Kohn (1999)
	ACT1Rd	CRTCGTACTCCTGCTTBGAGATCCAC	Groenewald et al. (2013)
*cmd*A	CAL-228F	GAGTTCAAGGAGGCCTTCTCCC	Carbone and Kohn 1999)
	CAL2Rd	TGRTCNGCCTCDCGGATCATCTC	Groenewald et al. (2013)
*his*3	CYLH3F	AGGTCCACTGGTGGCAAG	Crous et al. (2006)
	CYLH3R	AGCTGGATG TCCTTGGAC	
ITS	ITS5	GGAAGTAAAAGTCGTAACAAGG	White et al. (1990)
	ITS4	TCCTCCGCTTATTGATATGC	
LSU	LR0R	ACCCGCTGAACTTAAGC	Rehner and Samuels (1994)
	LR5	ATCCTGAGGGAAACTTC	Vilgalys and Hester (1990)
*rpb*1	RPB1-Ac	CAYCCWGGYTTYATCAAGAA	Castlebury et al. (2004)
	RPB1-Cr	CCNGCDATNTCRTTRTCCATRTA	
*rpb*2	RPB2-5F2	GGGGWGAYCAGAAGAAGGC	O’Donnell et al. (2007)
	RPB2-7cR	CCCATRGCTTGYTTRCCCAT	
*tef*1	EF1-728F	CATCGAGAAGTTCGAGAAGG	Carbone and Kohn (1999)
	EF2	GGARGTACCAGTSATCATG	O’Donnell et al. (1998)
*tub*2	T1	AACATGCGTGAGATTGTAAGT	O’Donnell and Cigelnik (1997)
	CYLTUB1R	AGTTGTCGG GACGGAAGAG	Crous et al. (2006)

### ﻿Sequence alignment and phylogenetic analyses

Based on BLAST searches in GenBank and recent publications (Bills et al. 2009; Gräfenhan et al. 2011; O’Donnell et al. 2012; Herrera et al. 2013; Dao et al. 2015, 2016; Lombard et al. 2015; Xu et al. 2021), using the large subunit of the ATP citrate lyase (*acl*1), actin (*act*) regions, calmodulin (*cmd*A), histone H3 (*his*3), the internal transcribed spacer (ITS), the partial large subunit nuclear rDNA (LSU), the RNA polymerase II largest subunit (*rpb*1), the RNA polymerase II second largest subunit (*rpb*2), translation elongation factor 1-alpha (*tef*1-α), β-tubulin (*tub*2) and sequence data, reference sequences were downloaded and separate phylogenetic analyses, based on single gene datasets were carried out to initially determine the placement of the two species. Information on the taxa used and GenBank accession numbers of our novel species are listed in Table 2. Alignments for the individual locus matrices were generated with the online version of MAFFT version 7.429 (Katoh and Standley 2013) and ambiguous regions were excluded using BioEdit version 7.0.5.3 (Hall 1999). Combined sequences of ITS, LSU, *tef*1-α, *rpb*1, *rpb*2, *acl*1, *act*, *tub*2, *cmd*A and *his*3 were performed by SequenceMatrix v.1.7.8 (Vaidya et al. 2011). Maximum Likelihood (ML) and Bayesian Inference (BI) were constructed as described in Xu et al (2020). The phylogenetic tree constructed was viewed and edited using FigTree version 1.4.2 and Adobe Illustrator CS6.

**Table 2.** Specimen information and GenBank accession numbers of the sequences used in this study.

Species	Strain/Voucher No.	GenBank Accession No.									
		acl1	act	cmdA	his3	ITS	LSU	rpb1	rpb2	tef l-α	tub2
*Cosmosporacoccinea*	CBS 341.70 ^T^	HQ897913	KM231221	KM231398	KM231550	HQ897827	KM231692	KM232242	HQ897777	KM231947	KM232086
*Cosmosporacymosa*	CBS 762.69 ^T^	HQ897914	KM231222	KM231399	KM231551	HQ897828	KM231693	KM232243	HQ897778	KM231948	KM232087
*Dialonectriaepisphaeria*	CBS 125494 = TG 2006-11	HQ897892	KM231227	KM231404	KM231556	HQ897811	KM231697	KM232248	HQ897756	KM231953	KM232092
*Dialonectriaullevolea*	CBS 125493 = TG 2007-56	HQ897918	KM231226	KM231403	KM231555	KM231821	KM231696	KM232247	HQ897782	KM231952	KM232091
*Fusicollaacetilerea*	BBA 63789 ^T^ = IMI181488 = NRRL20827	KM231065	–	–	–	HQ897790	U88108	–	HQ897701	–	–
*Fusicollaaquaeductuum*	CBS 837.85 = BBA 64559 = NRRL 20865	KM231067	–	KM231406	–	KM231823	KM231699	KM232250	HQ897744	KM231955	KM232094
*Fusicollaepistroma*	BBA 62201 ^T^ = IMI 85601 = NRRL 20439 = KNUF-20-PBU01	KM231069	–	–	–	OW982703	AF228352	LC592349	HQ897765	–	–
*Fusicollamatuoi*	CBS 581.78 = ATCC 18694 = MAFF 238445 = NRRL 20427	KM231070	KM231228	KM231405	KM231557	KM231822	KM231698	KM232249	HQ897720	KM231954	KM232093
*Macroconialeptosphaeriae*	CBS 717.74	KM231062	KM231236	KM231414	KM231564	KM231827	KM231707	KM232257	KM232390	JF735695	KM232099
*Macroconialeptosphaeriae*	CBS 100001 = CBS H-6030	KM231063	KM231234	KM231412	KM231562	HQ897810	KM231705	KM232255	HQ897755	KM231959	KM232097
*Macroconiapapilionacearum*	CBS 125495	HQ897912	KM231233	KM231411	KM231561	HQ897826	KM231704	KM232254	HQ897776	KM231958	KM232096
***Microcerachrysomphaludis***	**SICAUCC 22-0164** ^T^	**OQ569756**	**OQ569739**	**OQ599375**	**OQ569753**	**OQ434281**	**OQ434276**	**OQ569747**	**OQ569742**	**OQ438144**	**OQ569750**
***Microcerachrysomphaludis***	**SICAUCC 22-0165**	**OQ569757**	**OQ569740**	**OQ599376**	**OQ569754**	**OQ434282**	**OQ434277**	**OQ569748**	**OQ569743**	**OQ438145**	**OQ569751**
*Microceracoccophila*	CBS 310.34 ^T^ = NRRL 13962 = G.J.S. 98-50	HQ897843	KM231232	KM231410	KM231560	MH855540	KM231703	JX171462	JX171576	JF740692	KC291937
*Microceradiploa*	CBS 735.79 = BBA 62173 = NRRL 13966 = NRRL 36545	HQ897899	–	–	–	HQ897817	MW827663	JX171463	HQ897763	JF740693	–
*Microcerakuwanaspidis*	SICAUCC 21-0006 ^T^	MW462125	MW462126	MW462127	MW462128	MW484993	MW462905	MW462129	MW462124	MW462117	MW462130
*Microcerakuwanaspidis*	SICAUCC 21-0009	MZ044037	MZ044038	MZ044039	MZ044040	MZ029437	MZ029436	MZ044041	MZ044036	MZ044035	MZ044042
*Microceralarvarum*	CBS 169.30	HQ897855	–	–	EU860049	EU860064	EU860064	–	HQ897717	–	EU860025
*Microceralarvarum*	A.R. 4580 = CBS 133964	–	–	–	–	KC291751	KC291759	KC291894	–	KC291832	KC291935
*Microceralichenicola*	CPC 41114 ^T^ = CBS 149169	–	–	–	–	ON811502	ON811561	–	–	–	ON803591
*Microceraphysciae*	CPC 41284 = CBS 148283	–	–	–	–	OK664727	OK663766	OK651153	OK651168	OK651190	OK651208
*Microceraphysciae*	CPC 41038 ^T^ = CBS 148288	–	–	–	–	OK664728	OK663767	OK651154	OK651169	OK651191	OK651209
***Microcerapseudaulacaspidis***	**SICAUCC 22-0163** ^T^	**OQ569755**	**OQ569738**	**OQ599374**	**OQ569752**	**OQ434280**	**OQ434275**	**OQ569746**	**OQ569741**	**OQ438143**	**OQ569749**
*Microcerarubra*	CBS 638.76 ^T^ = BBA 62460 = NRRL 20475	HQ897903	KM231231	KM231409	EU860050	HQ897820	KM231702	KM232253	HQ897767	JF740696	EU860018
*Microcera* sp.	CPC 41230 = CBS 148313	–	–	–	–	ON811503	ON811562	ON803533	ON803543	ON803570	ON803592
*Pseudocosmosporaeutypae*	CBS 133966 ^T^ =A.R.4562	–	–	–	–	KC291721	KC291757	KC291871	–	KC291830	KC291912
*Pseudocosmosporaeutypellae*	C.H. 11-01 = CBS 133961 ^T^	–	–	–	–	KC291735	KC291766	KC291884	–	KC291837	KC291925
*Pseudocosmosporarogersonii*	CBS 133981 ^T^ = GJ.S. 90-56	–	–	–	–	KC291729	KC291780	KC291878	–	KC291852	KC291915
*Tilachlidiumbrachiatum*	CBS 505.67	KM231076	KM231249	KM231436	–	KM231839	KM231720	KM232272	KM232415	KM231976	KM232110
*Tilachlidiumbrachiatum*	CBS 363.97	KM231077	KM231248	KM231435	KM231583	KM231838	KM231719	KM232271	KM232414	KM231975	KM232109

Notes: superscript T represents ex-type or ex-epitype isolates. “-” means that the sequence is missing or unavailable. New sequences are listed in bold

### ﻿Genealogical concordance phylogenetic species recognition analysis

Phylogenetically closely-related species were analysed using the Genealogical Concordance Phylogenetic Species Recognition (GCPSR) model by performing a pairwise homoplasy index (PHI) test as described by Quaedvlieg et al. (2014). The PHI test was performed in SplitsTree v.4.17.1 (Huson 1998; Huson and Bryant 2006) in order to determine the recombination level within phylogenetically closely-related species using a 6-locus concatenated dataset (ITS, LSU, *tef*1-α, *acl*1, *cmd*A and *his*3). The results can be visualised by constructing a split graph using LogDet conversion and the Splits options. Pairwise homoplasy index below a 0.05 threshold (Ф_w_ < 0.05) indicates significant recombination present in the dataset. The relationship between closely-related species was visualised by constructing a Splits graph.

## ﻿Results

### ﻿Phylogenetic analyses

**Figure 1.**
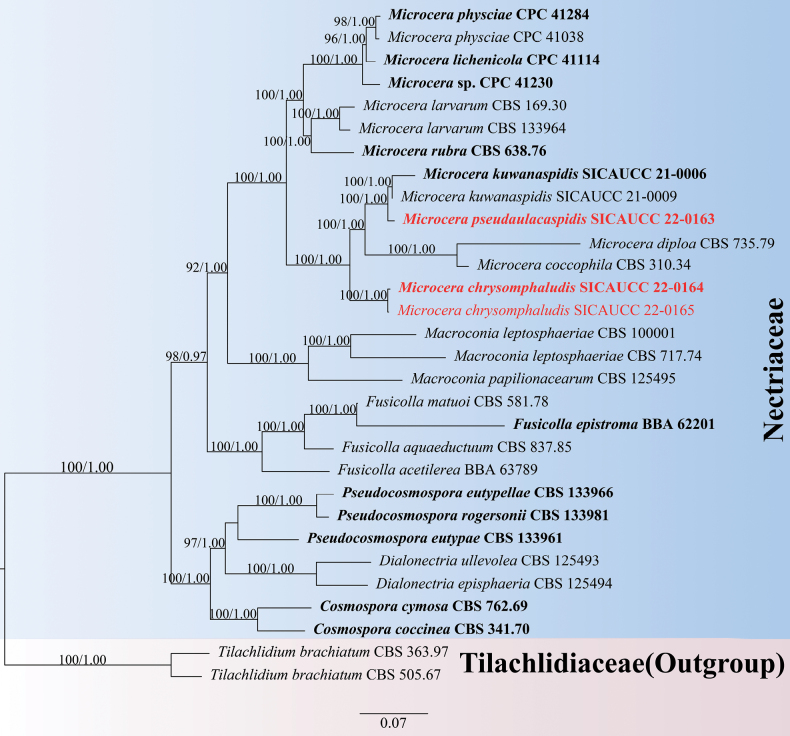
Phylogram generated from RAxML analysis, based on combined ITS, LSU, *tef*1-α, *rpb*1, r pb2, a cl1, *act*, *tub*2, *cmd*A and *his*3 sequence data of *Microcera* isolates. Bootstrap support values from Maximum Likelihood (MLBS, left) higher than 75% and Bayesian posterior probabilities (BIPP, right) equal to or greater than 0.95 are indicated at the nodes, respectively. The sequences from ex-type strains are in bold. The newly-generated sequence is in red.

The ML and BI analyses resulted in trees with similar topologies. Multi-locus phylogenetic analyses of species of Nectriaceae (Hypocreales) include sequences from 25 taxa and *Tilachlidiumbrachiatum* (Batsch) Petch (CBS 363.97, CBS 505.67) were used as outgroup (Fig. 1). The alignment contained 11882 characters (ITS = 1213, LSU = 1456, *tef*1-α = 1246, *rpb*1 = 1634, *rpb*2 = 2053, *acl*1 = 1060, *act* = 1206, *tub*2 = 707, *cmd*A = 779, *his*3 = 530), including gaps. The matrix had 4402 distinct alignment patterns, with 51.12% of undetermined characters or gaps. Estimated base frequencies were as follows: A = 0.236233, C = 0.270063, G = 0.255057, T = 0.238647, with substitution rates AC = 1.239837, AG = 3.452130, AT = 1.264349, CG = 0.971857, CT = 5.853200 and GT = 1.000000. The gamma distribution shape parameter α = 0.347827 and the Tree-Length = 2.637211. The best scoring RAxML tree with a final likelihood value of -68,438.855836 is presented in Fig. 1 where the isolates from this study formed two distinct, well-supported lineages (MLBS = 100%, BIPP = 1.00) and, thus, were considered to represent two previously-unknown species.

### ﻿Pairwise homoplasy index (PHI) test

**Figure 2.**
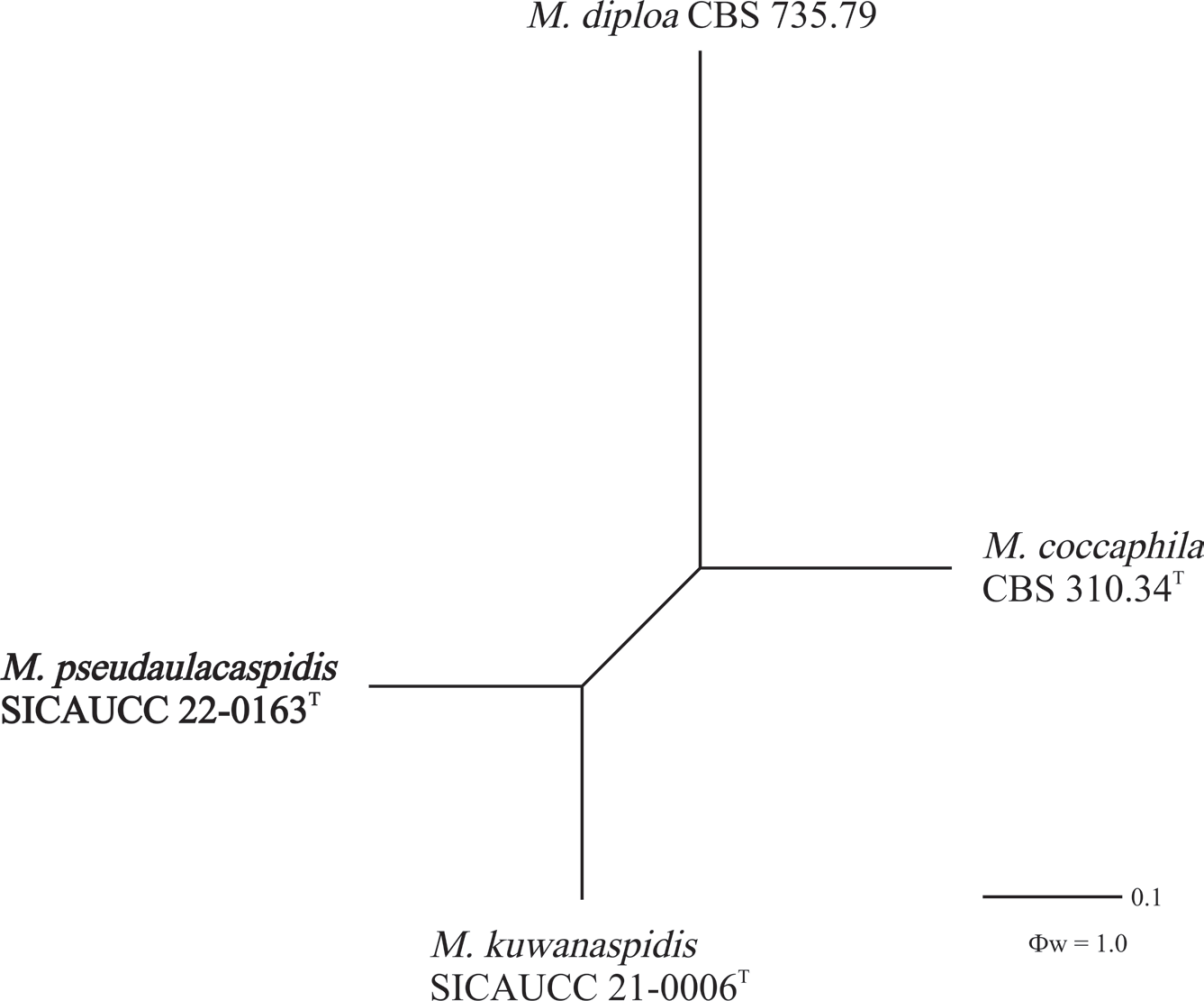
The result of the pairwise homoplasy index (PHI) test of closely-related species using both LogDet transformation and Splits decomposition. PHI test results (Ф_w_) < 0.05 indicate significant recombination within the dataset.

The pairwise homoplasy index (PHI) test revealed that there was no significant recombination (Ф_w_ = 1) between *Microcerapseudaulacaspidis* (SICAUCC 22-0163), *M.coccophila* (CBS 310.34), *M.diploa* (CBS 735.79) and *M.kuwanaspidis* (SICAUCC 21-0006) (Fig. 2).

### ﻿Taxonomy

#### ﻿ Microcera pseudaulacaspidis

Taxon classificationFungiHypocrealesNectriaceae

Feng Liu & C.L. Yang, sp. nov.

3CA458FD-B430-5122-B235-E65F475059EC

Index Fungorum No: 555034

Fig. 3

##### Etymology.

In reference to the generic name of scale insect from which it was isolated.

##### Holotype.

SICAU 22-0161.

##### Host.

*Pseudaulacaspispentagona* (Diaspididae, Homoptera)

##### Habitat.

On the trunk of *Juglansregia*.

##### Sexual state.

Undetermined.

##### Asexual state.

Stromata byssoid, well-developed, bright orange to orange-red, formed directly on the margin of host scales or their covers with 1–7 sporodochia. Sporodochia 250–900 μm long, 400–860 µm wide, (x–= 620 × 570 μm, n = 50), conical, orange-red, upright masses on margin of host scales. Macroconidia 70–120 µm long × 4.2–10.5 µm wide (x–= 95.7 × 6.5 μm, n = 50), hyaline or jasmine, cylindrical, slightly curved, slender towards each end, 3–10 septate, mostly 7–9 septate, difficult to distinguish apical cell and basal cell. Microconidia and chlamydospores were not observed.

**Figure 3.**
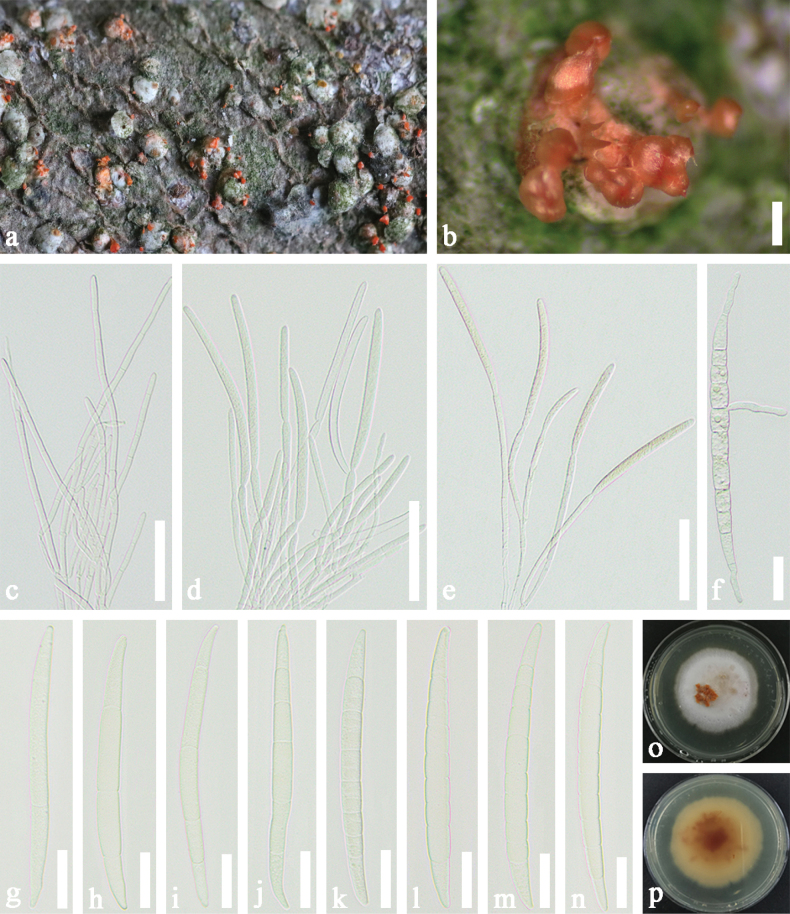
*Microcerapseudaulacaspidis* (SICAU 22-0161) **a, b** stromata and sporodochia on host substrate **c–e** conidiophore with developing macroconidia **f** germinated conidium **g–o** Macroconidia **o, p** colonies on PDA after 30 days. Scale bars: 200 µm (**a, b**); 20 µm (**c–e**); 10 µm (**f–n**).

##### Material examined.

China, Sichuan Province, Neijiang City, Dongxing District, Paifang Village walnut industrial base (29°48′15″N, 105°06′44″E, alt. 340 m), on scale insect *Pseudaulacaspispentagona*, 16 April 2022, Feng Liu, LF202204001, (SICAU 22-0161, holotype), ex-type culture SICAUCC 22-0163.

##### Culture characters.

Colonies from a single macroconidium on PDA grow slowly and reach approximately 2 cm in diameter after 12 days at 25 °C, circular, flat, producing masses of macroconidia in the centre of the colony, measuring 76–125 µm long × 5.3–7.6 µm wide (x–= 91.2 × 6.3 µm, n = 50), smaller than those in nature, white mycelium on the surface and the back of colonies is dark orange.

##### Notes.

Based on multi-gene phylogenetic analyses, *Microcerapseudaulacaspidis* is closely related to *M.kuwanaspidis* (Fig. 1). However, we observed significant differences in the DNA sequence data, including base-pair differences and gaps, with values of 1.45% (0 gaps), 17.67% (17 gaps), 3.22% (2 gaps), 1.53% (2 gaps), 1.70% (1 gap) and 3.82% (1 gap) in the ITS, LSU, *tef*1-α, *tub*2, *cmd*A and *his*3 genes, respectively. The PHI test also showed that no significant recombination events between *M.pseudaulacaspidis* and closely phylogenetically-related species occurred (Fig. 2). Based on a comparison of their morphological characteristics, *M.pseudaulacaspidis* can be distinguished from *M.kuwanaspidis* by shorter macroconidia (95.7 × 6.5 µm vs. 107 × 7.3 µm) with more septa (7–9-septate vs. 5–7-septate) (Xu et al. 2021). Given the morphological dissimilarities, distinct nucleotides at various sites and the well-supported lineage in our phylogeny, we have sufficient evidence to establish *M.pseudaulacaspidis* as a new species.

#### ﻿ Microcera chrysomphaludis

Taxon classificationFungiHypocrealesNectriaceae

Feng Liu & C.L. Yang, sp. nov.

54093027-5B77-5C71-BE7E-D36B03EEDF45

Index Fungorum No: 559445

Figs 4 , 5

##### Etymology.

In reference to the generic name of scale insect from which it was isolated.

##### Holotype.

SICAU 22-0162.

##### Host.

*Chrysomphalusaonidum* (Diaspididae, Homoptera)

##### Habitat.

On the trunk of *Juglansregia*.

##### Sexual state.

Perithecia 285–429 μm high, 216–386 µm diam. (x–= 350 × 290 μm, n = 50), scattered, gregarious, formed directly on margin of host scales, bright red to dark red, subglobose, ellipsoidal in section, a central, rounded, papillate ostiole, lined internally with periphyses. Peridium 62–95 µm thick, comprising two layers, outer stratum 32–55 µm thick, composed of small, hyaline to light brown cells of textura angularis; inner stratum 35–45 µm thick, composed of thinner, orange cells of textura angularis; thicker at sides towards apex, thinner at base. Hamathecium 8.5–19.2 µm diameter (x–= 12.3 µm, n = 30), longer than asci, septate, unbranched, paraphyses. Asci 83.3–128.5 × 7.5–15.2 µm (x–= 109.2 × 10.2 μm, n = 50), 8-spored, bitunicate, cylindrical, straight or curved, rounded at apex. Ascospores 16.8–27.5 × 7.8–10.8 µm (x–= 20.9 × 9.6 µm, n = 50), uniseriate, elliptical, with rounded ends, one-septate, slightly constricted at septum, hyaline, smooth-walled, with many guttules.

**Figure 4.**
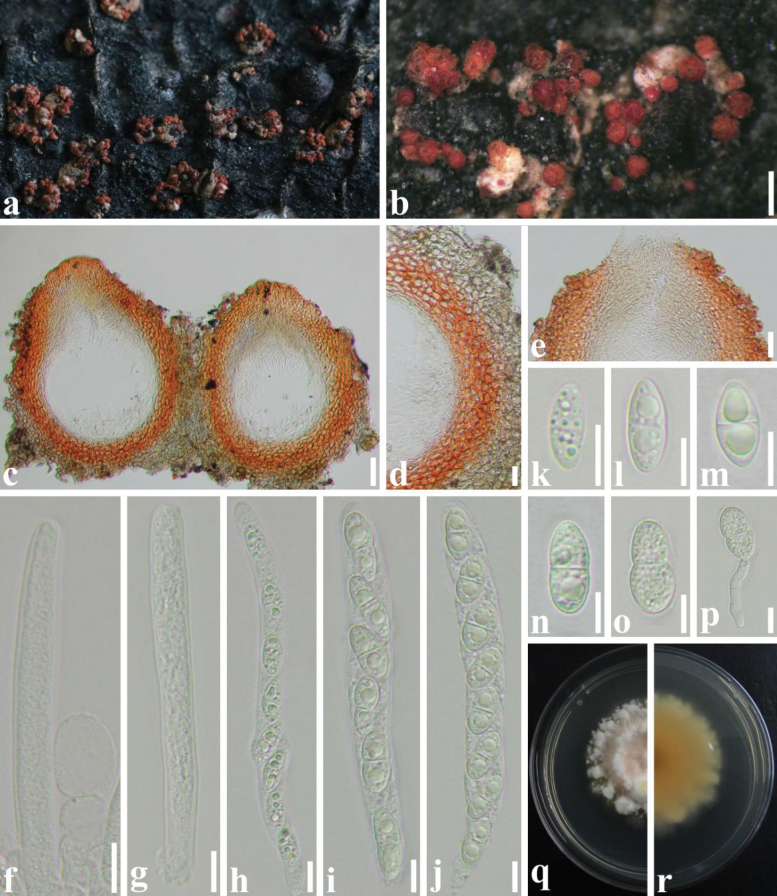
*Microcerachrysomphaludis* (SICAU 22-0162) **a, b** ascomata on host substrate **c** vertical section through ascostromata **d** peridium **e** ostiole of locule **f** paraphyses **h** ocular chamber **g–j** asci **k–o** ascospores **p** germinated ascospores; **q, r** colonies on PDA after 30 days. Scale bars: 200 µm (**a, b**); 50 µm **c**, 20 µm (**d, e**); 10 µm (**f**–**p**).

##### Asexual state.

Stromata byssoid, pale yellow, formed directly on margin of host scales with 1–6 sporodochia. Sporodochia conical, erupted, yellowish, scattered or aggregated. Macroconidia 73–89 long, 6.9–10.6 µm wide (x–= 78.8 × 8.5 μm, n = 50), hyaline, cylindrical, slightly curved, slender towards each end, 2–7 septa, mostly 4–6 septa, slightly constricted at septum, difficult to distinguish apical cell and basal cell. *Microconidia* and *chlamydospores* were not observed.

**Figure 5.**
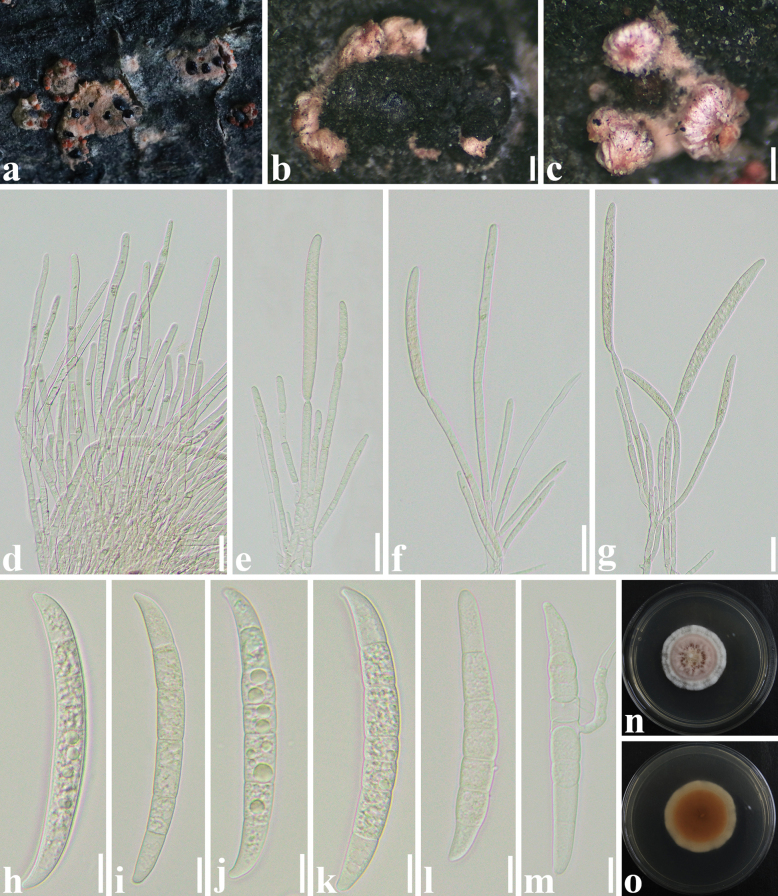
*Microcerachrysomphaludis* (SICAU 22-0163) **a–c** stromata and sporodochia on host substrate **d–g** conidiophore with developing macroconidia **h–l** macroconidia **m** germinated conidium **n, o** colonies on PDA after 30 days. Scale bars: 200 µm (**b, c**); 20 µm (**d–g**); 10 µm (**h–m**).

##### Material examined.

China, Sichuan Province, Liangshan Yi Autonomous Prefecture, Huili County (26°56′43″N, 107°16′16″E, alt. 1780 m), on scale insect *Chrysomphalusaonidum*, 8 October 2022, Feng Liu, LF202208001, (SICAU 22-0162, holotype), ex-type culture SICAUCC 22-0164. Ibid. LF202008002 (SICAU 22-0163, paratype), living culture SICAUCC 21-0165.

##### Culture characters.

Ascospores germinate on PDA within 12 h and cultures grow slowly on PDA. Colonies reach 2.4 cm in diameter after 20 days. Colonies from single conidia flocculent, clinging to medium, with irregular margin, white to pink mycelium on surface and back of colonies dark orange. Mycelium creamy-white starting at centre, but gradually becoming pale pink after 20 days, forming sparsely distributed mycelial clumps near edge of colony. Conidia germinate on PDA within 12 h, cultures grow slowly on PDA. Colonies 2.5 cm in diameter after 20 days. Colonies from single ascospores cottony and hard, with regular margin; mycelium creamy-white to pale pink, with concentric rings; back of colonies pale yellow.

##### Notes.

Multi-gene phylogenetic analyses have revealed that *Microcerachrysomphaludis* forms a highly robust clade that is closely related to *M.coccophila* and *M.diploa*. However, it is distinct from these two species with a high level of bootstrap support (ML/BY 100/1.00; Fig. 1). Morphologically, *M.chrysomphaludis* exhibits similar characteristics to *M.coccophila*, including superficial, subglobose, bright red ascomata, cylindrical asci and elliptical ascospores, as well as cylindrical macroconidia. However, *M.chrysomphaludis* can be differentiated from *M.coccophila* by its larger ascomata (285–429 × 216–386 µm vs. 194–387 × 194–355 μm), slightly shorter asci (109.2 × 10.2 μm vs. 115 × 15 µm), longer ascospores (16.8–27.5 × 7.8–10.8 μm vs. 14–19 × 6–10 μm) and shorter macroconidia (73–89 × 6.9–10.6 µm vs. 90–132 × 6–9 µm) and fewer septa (4–6 vs. 7–9) (Gräfenhan et al. 2011; Dao et al. 2015). Hence, we describe our collection as a new species in *Microcera*.

## ﻿Discussion

In this study, two new species (*Microcerachrysomphaludis* and *M.pseudaulacaspidis*) associated with scale insects from walnut were introduced, based on phylogenetic inferences of a combined ITS, LSU, *tef*1-α, *acl*1, *act*, *cmd*A, *his*3, *rpb*1, *rpb*2 and *tub*2 DNA sequence dataset and morphological evidence.

Ecologically, *Microcera* species are mainly distributed in tropical regions, but they have also been reported in the subtropical and temperate regions. Most of the *Microcera* species are pathogens of scale insects (Gräfenhan et al. 2011; O’Donnell et al. 2012; Dao et al. 2015, 2016; Crous et al. 2021a; Xu et al. 2021), However, two new species have recently been described from lichens (Crous et al. 2021b, 2022a). Most *Microcera* species infecting scale insects occur in the tree canopy and are more noticeable under moist conditions (Dao et al. 2015, 2016; Xu et al. 2021), consistent with the findings of this study. Morphologically, the sexual morph in this genus is characterised by orange to dark red perithecia with a blunt papilla producing cylindrical to narrowly clavate asci and 1(–3)-septate ascospores, while the asexual morph is predominantly fusarium-like, with verticillate to penicillate conidiophores producing small macroconidia (Gräfenhan et al. 2011; Lombard et al. 2015; Crous et al. 2021a, b). Similar morphs were observed and documented in this study to provide further evidence of a connection between our isolates and other *Microcera* species (e.g. Figs 4, 5).

Gräfenhan et al. (2011) analysed an association of *Microcera* to *Fusarium*, *Cladosterigma* Pat., *Mycogloea* L.S. Olive and *Tetracrium* Henn. and accepted four species in *Microcera*. In recent years, numerous newly-discovered species have been described by employing extensive sampling coupled with multigene phylogenies (Sung et al. 2007; Lombard and Crous 2012; Wei et al. 2019; Lucking et al. 2021). Lombard et al. (2015) performed a multi-gene phylogenetic analysis, using combined datasets of ITS, LSU, *tef*1-α, *acl*1, *act*, *cmd*A, *his*3, *rpb*1, *rpb*2 and *tub*2 to clarify intraspecific and intergeneric relationships within Nectriaceae. In this paper, *M.pseudaulacaspidis* was distinguished from *M.kuwanaspidis* and established as a new species, based on base-pair differences, particularly in the LSU (17.67%), *tef*1-α (3.22%) and *his*3 (3.82%). Additionally, *M.chrysomphaludis* formed a distinct and well-supported subclade and was found to be morphologically distinct from *M.coccophila* in terms of the size of asci, ascospores and macroconidia (Gräfenhan et al. 2011; O’Donnell et al. 2012; Dao et al. 2015). Through multigene phylogenetic analysis, the connection between the sexual and asexual morphs of *M.chrysomphaludis* was also confirmed.

Entomopathogenic fungi are common on scale insects and have great potential in biological control (Zha et al. 2019; Sharma et al. 2020). Based on field trials, *Microceralarvarum* has been reported to have a significant biological control effect of *Saissetiaoleae*, an economically important pest of olive and citrus plants (Cozzi et al. 2002). *Microcera* species have also been exploited for various biopharmaceuticals in recent years due to their secondary metabolites with medicinal properties. For instance, parnafungins, extracted from *M.larvarum*, have intrinsic antifungal activity (Parish et al. 2008). Isaka et al. (2015) isolated two new ascochlorin derivatives from cultures of *Microcera* sp. BCC 17074 and demonstrated their significant cytotoxic activities against various cancer cells. Furthermore, Cadelis et al. (2020) isolated four new secondary metabolites from *M.larvarum* isolates, which exhibited potent antimicrobial activity.

This paper presents novel findings of two new entomopathogenic fungi, *Microcerachrysomphaludis* and *M.pseudaulacaspidis*, which were isolated from scale insects found on walnut trees in China. We conducted surveys in numerous walnut orchards across Sichuan Province and observed significant infections of scale insects by these two species, resulting in high mortality rates, particularly in wet and humid conditions. Further screening and evaluation of these entomopathogenic fungi could facilitate their potential use as commercial biological control agents.

## Supplementary Material

XML Treatment for Microcera pseudaulacaspidis

XML Treatment for Microcera chrysomphaludis

## ﻿Additional information

### Author ORCIDs

Feng Liu https://orcid.org/0000-0003-4580-7169

Rajesh Jeewon https://orcid.org/0000-0002-8563-957X

Xiu-Lan Xu https://orcid.org/0000-0002-6832-5421

### Data availability

All of the data that support the findings of this study are available in the main text or Supplementary Information.

## References

[B1] Bills GF Platas G Overy DP Collado J Fillola A Jimenez MR Martin J Del VA Vicente F Tormo JR Pelaez F Calati K Harris G Parish C Xu D Roemer T (2009) Discovery of the parnafungins, antifungal metabolites that inhibit mrna polyadenylation, from the *Fusariumlarvarum* complex and other hypocrealean fungi. Mycologia 101(4): 449–472. 10.3852/08-163 19623926

[B2] Booth C (1971) The Genus *Fusarium*. Commonwealth Mycological Institute, Kew, Surrey, 237 pp.

[B3] Cadelis MM Geese S Gris L Weir BS Copp BR Wiles S (2020) A revised structure and assigned absolute configuration of theissenolactone A. Molecules (Basel, Switzerland) 25(20): 4823. 10.3390/molecules25204823 33092217 PMC7587954

[B4] Carbone I Kohn LM (1999) A method for designing primer sets for speciation studies in filamentous ascomycetes. Mycologia 3(3): 553–556. 10.1080/00275514.1999.12061051

[B5] Castlebury LA Rossman AY Sung GH Hyten AS Spatafora JW (2004) Multigene phylogeny reveals new lineage for *Stachybotryschartarum*, the indoor air fungus. Mycological Research 108(8): 864–872. 10.1017/S0953756204000607 15449591

[B6] Chomnunti P Hongsanan S Aguirre-Hudson B Tian Q Peršoh D Dhami MK Alias AS Xu J Liu X Stadler M Hyde KD (2014) The sooty moulds. Fungal Diversity 66: 1–36. 10.1007/s13225-014-0278-5

[B7] Cozzi G Stornelli C Moretti A Logrieco A Porcelli F (2002) Field evaluation of *Fusariumlarvarum* formulations in the biocontrol of *Saissetiaoleae* on olive in Apulia. Acta Horticulturae (586): 811–814. 10.17660/ActaHortic.2002.586.175

[B8] Crous PW Groenewald JZ Risède JM Simoneau P Hyde KD (2006) *Calonectria* species and their *Cylindrocladium* anamorphs: Species with clavate vesicles. Studies in Mycology 55: 213–226. 10.3114/sim.55.1.213 18490981 PMC2104717

[B9] Crous PW Lombard L Seifert KA Schroers HJ Gené J Guarro J Hirooka Y Kema GHJ Lamprecht SC Cai L Rossman AY Stadler M Summerbell RC Taylor JW Ploch S Visagie CM Frisvad JC Abdel-Azeem AM Abdollahzadeh J Abdolrasouli A Alberts JF Araújo JPM Bakhshi M Bendiksby M Bezerra JDP Boekhout T Câmara MPS Carbia M Cardinali G Castañeda-Ruiz RF Collemare J Croll D Damm U Decock CA de Vries RP Fan XL Groenewald JZ Guevara-Suarez M Gupta VK Guarnaccia V Hagen F Haelewaters D Hansen K Hashimoto A Hernández-Restrepo M Houbraken J Hubka V Hyde KD Iturriaga T Jeewon R Johnston PR Korsten L Kušan I Labuda R Lawrence DP Lee HB Lechat C Li HY Litovka YA Marin-Felix Y Matio Kemkuignou B Mctaggart AR Nakashima C Nilsson RH Noumeur SR Peralta MP Phillips AJL Pitt JI Polizzi G Quaedvlieg W Rajeshkumar KC Restrepo S Rhaiem A Robert J Robert V Salgado-Salazar C Samson RA Shivas RG Souza-Motta CM Sun GY Tan YP Taylor JE Tiago PV Váczy KZ van de Wiele N van der Merwe NA Verkley GJM Vieira WAS Weir BS Wijayawardene NN Yáñez-Morales MJ Yurkov A Zare R Zhang CL Thines M Sub MPP Sub EAB Microbiology M Physiology MP Biodiversity EA (2021a) *Fusarium*: More than a node or a foot-shaped basal cell. Studies in Mycology 98: 100116. 10.1016/j.simyco.2021.100116 PMC8379525 34466168

[B10] Crous PW Osieck ER Jurjevi Ž Boers J Van Iperen AL Starink-Willemse M Dima B Balashov S Bulgakov TS Johnston PR Morozova OV Pinruan U Sommai S Alvarado P Decock CA Lebel T Mcmullan-Fisher S Moreno G Shivas RG Zhao L Abdollahzadeh J Abrinbana M Ageev DV Akhmetova G Alexandrova AV Altés A Amaral AGG Angelini C Antonín V Arenas F Asselman P Badali F Baghela A Bañares A Barreto RW Baseia IG Bellanger JM Berraf-Tebbal A Biketova AY Bukharova NV Burgess TI Cabero J Câmara MPS Cano-Lira JF Ceryngier P Chávez R Cowan DA de Lima AF Oliveira RL Denman S Dang QN Dovana F Duarte IG Eichmeier A Erhard A Esteve-Raventós F Fellin A Ferisin G Ferreira RJ Ferrer A Finy P Gaya E Geering ADW Gil-Durán C Glässnerová K Glushakova AM Gramaje D Guard FE Guarnizo AL Haelewaters D Halling RE Hill R Hirooka Y Hubka V Iliushin VA Ivanova DD Ivanushkina NE Jangsantear P Justo A Kachalkin AV Kato S Khamsuntorn P Kirtsideli IY Knapp DG Kochkina GA Koukol O Kovács GM Kruse J Kumar TKA Kušan I Læssøe T Larsson E Lebeuf R Levicán G Loizides M Marinho P Luangsa-Ard JJ Lukina EG Magaña-Dueñas V Maggs-Kölling G Malysheva EF Malysheva VF Martín B Martín MP Matočec N Mctaggart AR Mehrabi-Koushki M Mešić A Miller AN Mironova P Moreau PA Morte A Müller K Nagy LG Nanu S Navarro-Ródenas A Nel WJ Nguyen TH Nóbrega TF Noordeloos ME Olariaga I Overton BE Ozerskaya SM Palani P Pancorbo F Papp V Pawłowska J Pham TQ Phosri C Popov ES Portugal A Pošta A Reschke K Reul M Ricci GM Rodríguez A Romanowski J Ruchikachorn N Saar I Safi A Sakolrak B Salzmann F Sandoval-Denis M Sangwichein E Sanhueza L Sato T Sastoque A Senn-Irlet B Shibata A Siepe K Somrithipol S Spetik M Sridhar P Stchigel AM Stuskova K Suwannasai N Tan YP Thangavel R Tiago I Tiwari S Tkalčec Z Tomashevskaya MA Tonegawa C Tran HX Tran NT Trovão J Trubitsyn VE Van Wyk J Vieira WAS Vila J Visagie CM Vizzini A Volobuev SV Vu DT Wangsawat N Yaguchi T Ercole E Ferreira BW de Souza AP Vieira BS Groenewald JZ (2021b) Fungal planet description sheets: 1284-1382. Persoonia 47: 178–374. 10.3767/persoonia.2021.47.06 PMC10486635 37693795

[B11] Crous PW Begoude BAD Boers J Braun U Declercq B Dijksterhuis J Elliott TF Garay-Rodriguez GA Jurjević Ž Kruse J Linde CC Loyd A Mound L Osieck ER Rivera-Vargas LI Quimbita AM Rodas CA Roux J Schumacher RK Starink-Willemse M Thangavel R Trappe JM van Iperen AL Van Steenwinkel C Wells A Wingfield MJ Yilmaz N Groenewald JZ (2022a) New and interesting fungi. 5. Fungal Systematics and Evolution 10(1): 19–90. 10.3114/fuse.2022.10.02 36789279 PMC9903348

[B12] Crous PW Sandoval-Denis M Costa MM Groenewald JZ van Iperen AL Starink-Willemse M Hernández-Restrepo M Kandemir H Ulaszewski B de Boer W Abdel-Azeem AM Abdollahzadeh J Akulov A Bakhshi M Bezerra JDP Bhunjun CS Câmara MPS Chaverri P Vieira WAS Decock CA Gaya E Gené J Guarro J Gramaje D Grube M Gupta VK Guarnaccia V Hill R Hirooka Y Hyde KD Jayawardena RS Jeewon R Jurjević Ž Korsten L Lamprecht SC Lombard L Maharachchikumbura SSN Polizzi G Rajeshkumar KC Salgado-Salazar C Shang QJ Shivas RG Summerbell RC Sun GY Swart WJ Tan YP Vizzini A Xia JW Zare R González CD Iturriaga T Savary O Coton M Coton E Jany JL Liu C Zeng ZQ Zhuang WY Yu ZH Thines M (2022b) *Fusarium* and allied fusarioid taxa (FUSA). 1. Fungal Systematics and Evolution 9(1): 161–200. 10.3114/fuse.2022.09.08 35978986 PMC9355104

[B13] Dai DQ Phookamsak R Wijayawardene NN Li WJ Bhat DJ Xu JC Taylor JE Hyde KD Chukeatirote E (2016) Bambusicolous fungi. Fungal Diversity 82(1): 1–105. 10.1007/s13225-016-0367-8

[B14] Dao HT Beattie GAC Rossman AY Burgess LW Holford P (2015) Systematics and biology of two species of *Microcera* associated with armoured scales on citrus in Australia. Mycological Progress 14(4): 1–14. 10.1007/s11557-015-1044-0

[B15] Dao HT Beattie GAC Rossman AY Burgess LW Holford P (2016) Four putative entomopathogenic fungi of armoured scale insects on citrus in Australia. Mycological Progress 15(5): 47. 10.1007/s11557-016-1188-6

[B16] Gräfenhan T Schroers HJ Nirenberg HI Seifert KA (2011) An overview of the taxonomy, phylogeny, and typification of nectriaceous fungi in *Cosmospora*, *Acremonium*, *Fusarium*, *Stilbella*, and *Volutella*. Studies in Mycology 68: 79–113. 10.3114/sim.2011.68.04 21523190 PMC3065986

[B17] Groenewald JZ Nakashima C Nishikawa J Shin HD Park JH Jama AN Groenewald M Braun U Crous PW (2013) Species concepts in *Cercospora*: Spotting the weeds among the roses. Studies in Mycology 75: 115–170. 10.3114/sim0012. 10.3114/sim0012 24014899 PMC3713887

[B18] Hall TA (1999) BioEdit: A user-friendly biological sequence alignment editor and analysis program for Windows 95/98/NT. Nucleic Acids Symposium Series 41: 95–98.

[B19] Herrera CS Rossman AY Samuels GJ Chaverri P (2013) *Pseudocosmospora*, a new genus to accommodate *Cosmosporavilior* and related species. Mycologia 105(5): 1287–1305. 10.3852/12-395 23921243

[B20] Huson DH (1998) SplitsTree: Analyzing and visualizing evolutionary data. Bioinformatics 14(1): 68–73. 10.1093/bioinformatics/14.1.68 9520503

[B21] Huson DH Bryant D (2006) Application of phylogenetic networks in evolutionary studies. Molecular Biology and Evolution 23(2): 254–267. 10.1093/molbev/msj030 16221896

[B22] Isaka M Yangchum A Supothina S Laksanacharoen P Jennifer LJ Hywel-Jones NL (2015) Ascochlorin derivatives from the leafhopper pathogenic fungus *Microcera* sp. BCC 17074. The Journal of Antibiotics 68(1): 47–51. 10.1038/ja.2014.90 24984794

[B23] Jeewon R Hyde KD (2016) Establishing species boundaries and new taxa among fungi:recommendations to resolve taxonomic ambiguities. Mycosphere : Journal of Fungal Biology 7(11): 1669–1677. 10.5943/mycosphere/7/11/4

[B24] Katoh K Standley DM (2013) Mafft multiple sequence alignment software version 7: Improvements in performance and usability. Molecular Biology and Evolution 30(4): 772–780. 10.1093/molbev/mst010 23329690 PMC3603318

[B25] Leslie JF Summerell BA (2006) The *Fusarium* Laboratory Manual, 1^st^ edn. Blackwell Publisher, Ames, IA, USA, 369 pp. 10.1002/9780470278376

[B26] Lombard L Crous PW (2012) Phylogeny and taxonomy of the genus *Gliocladiopsis*. Persoonia 28(1): 25–33. 10.3767/003158512X635056 23105151 PMC3409413

[B27] Lombard L Merwe NA Groenewald JZ Crous PW (2015) Generic concepts in Nectriaceae. Studies in Mycology 80(1): 189–245. 10.1016/j.simyco.2014.12.002 26955195 PMC4779799

[B28] Lucking R Aime MC Robbertse B Miller AN Aoki T Ariyawansa HA Cardinali G Crous PW Druzhinina IS Geiser DM Hawksworth DL Hyde KD Irinyi L Jeewon R Johnston PR Kirk PM Malosso E May TW Meyer W Nilsson HR Opik M Robert V Stadler M Thines M Vu D Yurkov AM Zhang N Schoch CL (2021) Fungal taxonomy and sequence-based nomenclature. Nature Microbiology 6(5): 540–548. 10.1038/s41564-021-00888-x PMC10116568 33903746

[B29] Nelson PE Toussoun TA Marasas WFO (1983) *Fusarium* species: An Illustrated Manual for Identification; Pennsylvania State University Press, University Park, PA, USA, 193 pp.

[B30] O’Donnell K Cigelnik E (1997) Two divergent intragenomic rDNA ITS2 types within a monophyletic lineage of the fungus *Fusarium* are nonorthologous. Molecular Phylogenetics and Evolution 7(1): 103–116. 10.1006/mpev.1996.0376 9007025

[B31] O’Donnell K Kistler HC Cigelnik E Ploetz RC (1998) Multiple evolutionary origins of the fungus causing Panama disease of banana: Concordant evidence from nuclear and mitochondrial gene genealogies. Proceedings of the National Academy of Sciences of the United States of America 95(5): 2044–2049. 10.1073/pnas.95.5.2044 9482835 PMC19243

[B32] O’Donnell K Sarver BA Brandt M Chang DC Noble-Wang J Park BJ Sutton DA Benjamin L Lindsley M Padhye A Geiser DM Ward TJ (2007) Phylogenetic diversity and microsphere array-based genotyping of human pathogenic Fusaria, including isolates from the multistate contact lens-associated U.S. keratitis outbreaks of 2005 and 2006. Journal of Clinical Microbiology 45(7): 2235–2248. 10.1128/JCM.00533-07 17507522 PMC1933018

[B33] O’Donnell K Humber RA Geiser DM Kang S Park B Robert VA Crous PW Johnston PR Aoki T Rooney AP Rehner SA (2012) Phylogenetic diversity of insecticolous fusaria inferred from multilocus DNA sequence data and their molecular identification via FUSARIUM-ID and *Fusarium MLST.* Mycologia 104(2): 427–445. 10.3852/11-179 22086911

[B34] Parish CA Smith SK Calati K Zink D Wilson K Roemer T Jiang B Xu D Bills G Platas G Pelaez F Diez MT Tsou N Mckeown AE Ball RG Powles MA Yeung L Liberator P Harris G (2008) Isolation and structure elucidation of parnafungins, antifungal natural products that inhibit mRNA polyadenylation. Journal of the American Chemical Society 130(22): 7060–7066. 10.1021/ja711209p 18461935

[B35] Quaedvlieg W Binder M Groenewald JZ Summerell BA Carnegie AJ Burgess TI Crous PW (2014) Introducing the consolidated species concept to resolve species in the Teratosphaeriaceae. Persoonia 33(1): 1–40. 10.3767/003158514X681981 25737591 PMC4312929

[B36] Rehner SA Samuels GJ (1994) Taxonomy and phylogeny of *Gliocladium* analysed from nuclear large subunit ribosomal DNA sequences. Mycological Research 98(6): 625–634. 10.1016/S0953-7562(09)80409-7

[B37] Senanayake IC Rathnayake AR Marasinghe DS Calabon MS Gentekaki E Lee HB Hurdeal VG Pem D Dissanayake LS Wijesinghe SN Bundhun D Nguyen TT Goonasekara ID Abeywickrama PD Bhunjun CS Jayawardena RS Wanasinghe DN Jeewon R Bhat DJ Mm X (2020) Morphological approaches in studying fungi: Collection, examination, isolation, sporulation and preservation. Mycosphere : Journal of Fungal Biology 11(1): 2678–2754. 10.5943/mycosphere/11/1/20

[B38] Sharma A Srivastava A Shukla AK Srivastava K Srivastava AK Saxena AK (2020) Entomopathogenic fungi: a potential source for biological control of insect pests. Springer, Singapore, 225–250. 10.1007/978-981-15-3151-4_9

[B39] Sung GH Sung JM Hywei-Jones ML Spatafora JW (2007) A multi-gene phylogeny of clavicipitaceae (Ascomycota, Fungi): Identification of localized incongruence using a combinational bootstrap approach. Molecular Phylogenetics and Evolution 44(3): 1204–1223. 10.1016/j.ympev.2007.03.011 17555990

[B40] Vaidya G Lohman DJ Meier R (2011) SequenceMatrix: Concatenation software for the fast assembly of multi-gene datasets with character set and codon information. Cladistics 27(2): 171–180. 10.1111/j.1096-0031.2010.00329.x 34875773

[B41] Vilgalys R Hester M (1990) Rapid genetic identification and mapping of enzymatically amplified ribosomal DNA from several *Cryptococcus* species. Journal of Bacteriology 172(8): 4238–4246. 10.1128/jb.172.8.4238-4246.1990 2376561 PMC213247

[B42] Wanasinghe DN Mortimer PE Xu J (2021) Insight into the systematics of microfungi colonizing dead woody twigs of *Dodonaeaviscosa* in Honghe (China). Journal of Fungi (Basel, Switzerland) 7(3): 180. 10.3390/jof7030180 33802406 PMC7999967

[B43] Wei D Wanasinghe DN Hyde KD Mortimer PE Xu J Xiao Y Bhunjun CS To-Anun C (2019) The genus *Simplicillium*. MycoKeys 60: 69–92. 10.3897/mycokeys.60.38040 31798310 PMC6879665

[B44] White TJ Bruns T Lee S Taylor J (1990) Amplification and direct sequencing of fungal ribosomal RNA genes for phylogenetics. In: Innis MA, Gelfand DH, Sninsky JJ, White TJ (Eds) PCR protocols: a guide to methods and applications, Academic Press, San Diego, California, 315–322. 10.1016/B978-0-12-372180-8.50042-1

[B45] Xu XL Yang CL Jeewon R Wanasinghe DN Liu YG Xiao QG (2020) Morpho-molecular diversity of Linocarpaceae (Chaetosphaeriales): *Claviformispora* gen. nov. from decaying branches of *Phyllostachysheteroclada*. MycoKeys 70: 1–17. 10.3897/mycokeys.70.54231 32742178 PMC7381431

[B46] Xu XL Zeng Q Lv YC Jeewon R Maharachchikumbura S Wanasinghe DN Hyde KD Xiao QG Liu YG Yang CL (2021) Insight into the systematics of novel entomopathogenic fungi associated with armored scale insect, *Kuwanaspishowardi* (Hemiptera: Diaspididae) in China. Journal of Fungi (Basel, Switzerland) 7(8): 628. 10.3390/jof7080628 34436167 PMC8401669

[B47] Zha LS Wen TC Jeewon R Xie ZM Boonmee S Eungwanichayapant PD Hyde KD (2019) Xuefeng Cordyceps: Insights into species diversity, life cycle and host association. Current Science 117(5): 839–846. 10.18520/cs/v117/i5/839-846

